# Supplementary material for: Biodegradable Poly(D-L-lactide-co-glycolide) (PLGA)-Infiltrated Bioactive Glass (CAR12N) Scaffolds Maintain Mesenchymal Stem Cell Chondrogenesis for Cartilage Tissue Engineering
Source: Cells. 2022 May 7;11(9):1577. doi: 10.3390/cells11091577 (PMC9100331; doi:10.3390/cells11091577)
Supplement: Supplementary file 1 [file cells-11-01577-s001.zip › cells-1639117-supplementary.pdf]

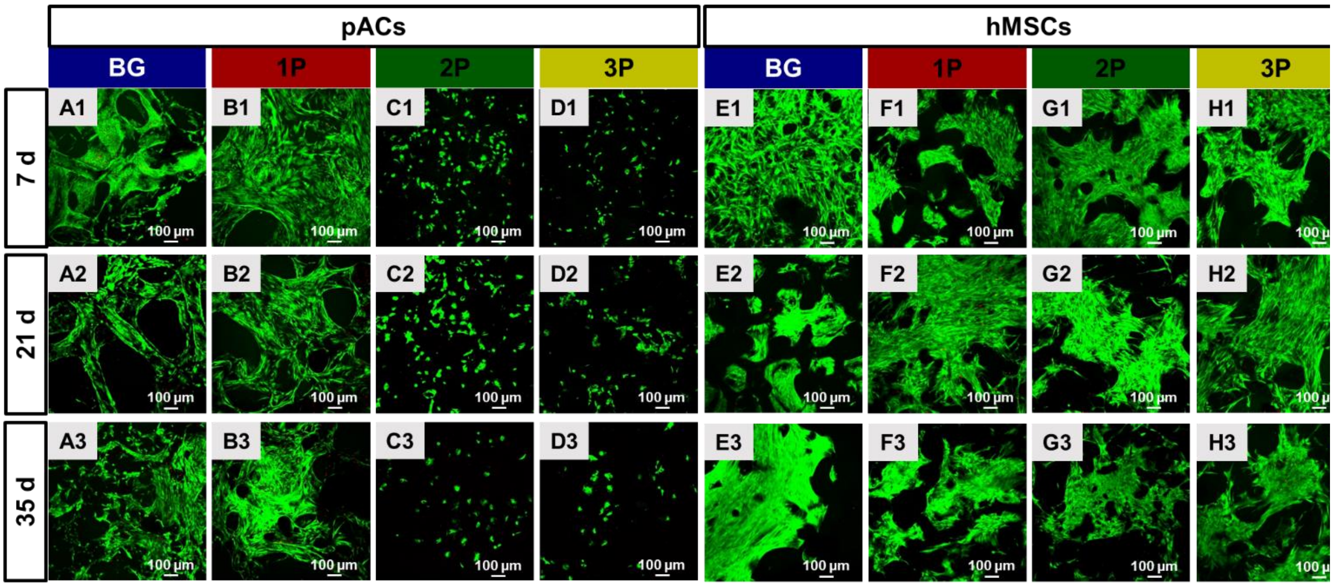

Supplemental Figure S1 **Viability assay of colonized scaffolds**. Representative pictures of three independent experiments show vital (green) and dead (red) porcine articular chondrocytes (pACs) (**A1-D3**) and human mesenchymal stromal cells (hMSCs) (**E1 – H3**) over a cultivation time of 7 to 35 days. Three independent experiments (n=3) with cells from three different donors were performed. Scale bar: 100 µm

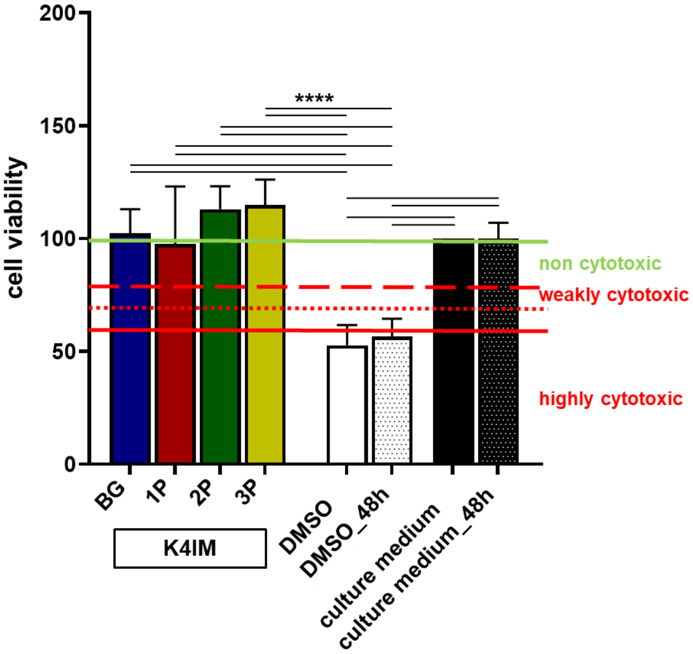

Supplemental Figure S2 **Cytotoxicity assay using BG (blue bars), 1P (red bars), 2P (green bars) and 3P (yellow bars) scaffold extracts**. Viability of synovial fibroblast cell line K4IM treated for 24 hours with scaffold extracts (48 hours

extraction) from three different scaffold charges. Cell viability was above 90% in cells treated with all scaffold extracts. Cell viability of the positive control with 10% dimethylsulfoxide (DMSO, white bar) and 10% DMSO mixed with 48 hours incubated culture medium (white bar with black dots) was below 70% and that of the negative control with culture medium (black) was 100% and the negative control with 48 hours incubated culture medium (black with white dots). The CellTiter 96®Aqueous One Solution Cell Proliferation Assay was used to assess cytotoxicity. n=3. Ordinary one-way ANOVA with multiple comparison, p values: **** < 0.0001.
